# Blastomycosis Mortality Rates, United States, 1990–2010

**DOI:** 10.3201/eid2011.131175

**Published:** 2014-11

**Authors:** Diana Khuu, Shira Shafir, Benjamin Bristow, Frank Sorvillo

**Affiliations:** University of California, Los Angeles, California, USA (D. Khuu, S. Shafir, F. Sorvillo);; Icahn School of Medicine at Mount Sinai, New York, New York, USA (B. Bristow)

**Keywords:** Blastomyces dermatitidis, conidia, airborne, yeast, blastomycosis, endemic mycoses, mycosis, mortality, fungi

## Abstract

Blastomycosis is a potentially fatal fungal infection endemic to parts of North America. We used national multiple-cause-of-death data and census population estimates for 1990–2010 to calculate age-adjusted mortality rates and rate ratios (RRs). We modeled trends over time using Poisson regression. Death occurred more often among older persons (RR 2.11, 95% confidence limit [CL] 1.76, 2.53 for those 75–84 years of age vs. 55–64 years), men (RR 2.43, 95% CL 2.19, 2.70), Native Americans (RR 4.13, 95% CL 3.86, 4.42 vs. whites), and blacks (RR 1.86, 95% CL 1.73, 2.01 vs. whites), in notably younger persons of Asian origin (mean = 41.6 years vs. 64.2 years for whites); and in the South (RR 18.15, 95% CL 11.63, 28.34 vs. West) and Midwest (RR 23.10, 95% CL14.78, 36.12 vs. West). In regions where blastomycosis is endemic, we recommend that the diagnosis be considered in patients with pulmonary disease and that it be a reportable disease.

## Abstract 2

Risks for illness and death caused by the *Blastomyces dermatitidis* fungus are affected by demographic, geographic, and behavioral factors.

Blastomycosis is a systemic infection caused by the thermally dimorphic fungus *Blastomyces dermatitidis* that can result in severe disease and death among humans and animals. *B. dermatitidis* is endemic to the states bordering the Mississippi and Ohio Rivers, the Great Lakes, and southern Canada; it is found in moist, acidic, enriched soil near wooded areas and in decaying vegetation or other organic material (*1*). Conidia, the spores, become airborne after disruption of areas contaminated with *B. dermatitidis*. Infection occurs primarily through inhalation of the *B. dermatitidis* spores into the lungs, where they undergo transition to the invasive yeast phase. The infection can progress in the lung, where the infection may be limited, or it can disseminate and result in extrapulmonary disease, affecting other organ systems (*2*).

The incubation period for blastomycosis is 3–15 weeks. About 30%–50% of infections are asymptomatic. Pulmonary symptoms are the most common clinical manifestations; however, extrapulmonary disease can frequently manifest as cutaneous and skeletal disease and, less frequently, as genitourinary or central nervous system disease. Liver, spleen, pericardium, thyroid, gastrointestinal tract, or adrenal glands may also be involved (*3*). Misdiagnoses and delayed diagnoses are common because the signs and symptoms resemble those of other diseases, such as bacterial pneumonia, influenza, tuberculosis, other fungal infections, and some malignancies (*4*). Accurate diagnosis relies on a high index of suspicion with confirmation by using histologic examination, culture, antigen detection assays, or PCR tests (*5*).

Antifungal agents, such as itraconazole for mild or moderate disease and amphotericin B for severe disease, can provide effective therapy, especially when administered early (*1*,*2*). With appropriate treatment, blastomycosis can be successfully treated without relapse; however, case-fatality rates of 4%–22% have been observed (*4*,*6*–*9*). Although spontaneous recovery can occur (*10*,*11*), case-patients often require monitoring of clinical progress and administration of drugs on an inpatient basis. Previous studies estimated average hospitalization costs for adults to be $20,000; that is likely less than the current true cost (*12*). Some reviews of outbreaks indicate a higher distribution of infection among persons of older age, male sex (*2*,*13*), black, Asian, and Native American racial/ethnic groups (*3*,*13*), and those who have outdoor occupations (*13*,*14*). Both immunocompetent and immunocompromised hosts may experience disease and death (*2*,*6*,*15**–**19*), although *B. dermatitidis* disproportionately affects immunocompromised patients, who tend to have more rapid and extensive pulmonary involvement, extrapulmonary infection, complications, and higher mortality rates (25%–54%) (*2*,*6*,*16*–*19*).

Past studies have expanded the knowledge about blastomycosis through focusing on cases documented in specific immunocompromised persons and statewide occurrences or in areas in which the disease is endemic (*4*,*6*–*9*,*16*–*18*); however, such studies may be limited for making definitive conclusions by their scope and small sample size. Much remains unknown about the public health burden of blastomycosis-related deaths in the United States. Reports suggest an increase in the number of blastomycosis cases in recent years (*13*,*20*). Clearer identification of risk factors from national data may raise awareness of blastomycosis in the United States and support adding it to the list of reportable diseases in regions where the pathogen is endemic to improve surveillance and control. In this study, we assessed the public health burden of blastomycosis-related deaths by examining US mortality-associated data and evaluating demographic, temporal, and geographic associations as potential risk factors.

## Methods

### Data Source

We used publicly available multiple-cause-of-death (MCOD) data from the National Center for Health Statistics to examine blastomycosis-related deaths in the United States during 1990–2010. These data are derived from US death certificates and include information on the causes of death coded by the International Classification of Diseases, 9th and 10th Revisions (ICD-9, ICD-10), demographic variables of age, sex, and race/ethnicity, date of death, and geographic region of residence.

### Case Definition

We defined a case-patient as deceased US resident listed in the MCOD dataset during 1990–2010 whose death certificate listed blastomycosis as the underlying or contributing cause of death. The ICD-9 code 116.0 (years 1990–1998) and ICD-10 codes B40.0–B40.9 (years 1999–2010) were used to identify blastomycosis-related deaths.

### Analysis

To ensure more stable estimates, we aggregated data for the study period. We calculated mortality rates and rate ratios (RRs) with 95% confidence limits (CLs) by age, sex, race/ethnicity, geographic region, and year of death using a maximum likelihood analysis presuming the response variable had a Poisson distribution (*21*), and with bridged-race population estimates data and designated geographic boundaries from the US census. We computed age-adjusted mortality rates using adjustment weights from the year 2000 US standard population data. We assessed temporal trends in age-adjusted mortality rates using a Poisson regression model of deaths per person-years in the population, designating year and age group dummy variables as independent variables, and the population as the offset. We calculated the percentage change by year based on the estimated slope parameter and examined the Poisson regression models for overdispersion. We performed all analyses using SAS for Windows version 9.4 (SAS Institute Inc., Cary, NC, USA).

## Results

We identified 1,216 blastomycosis-related deaths among 49,574,649 deaths in the United States during 1990–2010. Among those 1,216 deaths, blastomycosis was reported as the underlying cause of death for 741 (60.9%), and as a contributing cause of death for 475 (39.1%). The overall age-adjusted mortality rate for the period was 0.21 (95% CL 0.20, 0.22) per 1 million person-years. Using Poisson regression, we identified a 2.21% (95% CL −3.11, −1.29) decline in blastomycosis-related mortality rates during the period (Figure).

**Figure:**
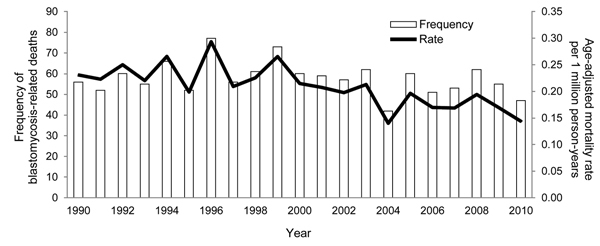
Number of blastomycosis-related deaths and age-adjusted mortality rates per 1 million person-years, by year, United States, 1990–2010.

### Age

The mean age at death from blastomycosis was 60.8 years. Using 75 as the average age at death (*22*,*23*), we calculated that 19,097 years of potential life were lost. The mortality rates associated with blastomycosis increased with increasing age, peaking in the 75- to 84-year age group (Table 1). The mean age at death from blastomycosis was significantly lower among Hispanics (p<0.01), Native Americans (p<0.01), blacks (p<0.01), and Asians (p<0.01) than among whites based on the *t* test for difference in means.

**Table 1:** Blastomycosis-related deaths by sex, race/ethnicity, age group, and geographic region with corresponding age-adjusted mortality rates, United States, 1990–2010*

Characteristic	No. (%) deaths	Mean age at death, y	Age-adjusted mortality rate/1 million person-years (95% CL)†	Age-adjusted mortality rate ratio (95% CL)
Sex				
F	409 (33.6)	62.3	0.14 (0.13, 0.16)	1
M	807 (66.4)	60.1	0.35 (0.32, 0.37)	2.43 (2.19, 2.70)
Race/ethnicity				
White	918 (75.5)	64.2	0.22 (0.21, 0.23)	1
Hispanic	25 (2.1)	53.0	0.06 (0.03, 0.08)	0.25 (0.19, 0.33)
Black	223 (18.3)	50.6	0.41 (0.35, 0.46)	1.86 (1.73, 2.01)
Asian	20 (1.6)	41.6	0.11 (0.06, 0.15)	0.47 (0.41, 0.55)
Native American	30 (2.5)	52.9	0.91 (0.57, 1.25)	4.13 (3.86, 4.42)
Age, y‡				
<1	1 (0.1)	NA	0.01 (0.00, 0.04)	0.03 (0.00, 0.19)
1–4	1 (0.1)	NA	0.00 (0.00, 0.01)	0.01 (0.00, 0.05)
5–14	6 (0.5)	9.2	0.01 (0.00, 0.01)	0.02 (0.01, 0.04)
15–24	33 (2.7)	19.3	0.04 (0.03, 0.05)	0.09 (0.06, 0.13)
25–34	59 (4.9)	30.3	0.07 (0.05, 0.09)	0.16 (0.12, 0.21)
35–44	122 (10.0)	39.7	0.14 (0.11, 0.16)	0.31 (0.25, 0.39)
45–54	189 (15.5)	49.7	0.25 (0.21, 0.28)	0.56 (0.46, 0.68)
55–64	240 (19.7)	59.7	0.44 (0.38. 0.49)	1
65–74	257 (21.1)	69.9	0.65 (0.57, 0.73)	1.48 (1.24, 1.76)
75–84	235 (19.3)	78.9	0.92 (0.80, 1.04)	2.11 (1.76, 2.53)
≥85	73 (6.0)	88.1	0.81 (0.62, 0.99)	1.84 (1.42, 2.40)
Geographic region‡				
West	20 (1.7)	67.1	0.02 (0.01, 0.02)	1
South	643 (52.9)	60.5	0.31 (0.28, 0.33)	18.15 (11.63, 28.34)
Midwest	533 (43.8)	61.1	0.39 (0.36, 0.42)	23.10 (14.78, 36.12)
Northeast	20 (1.7)	61.6	0.02 (0.01, 0.02)	1.00 (0.54, 1.86)
Total	1,216 (100)	60.8	0.21 (0.20, 0.22)	

*CL, confidence limit; NA, not applicable. States in western region: AK, AZ, CA, CO, HI, ID, MT, NM, NV, OR, UT, WA, WY. States in southern region: AL, AR, DC, DE, FL, GA, KY, LA, MS, NC, OK, SC, TN, TX, VA, WV. States in midwestern region: IA, IL, IN, KS, MI, MN, MO, MS, NE, ND, OH, SD, WI. States in northeastern region: CT, MA, ME, NH, NJ, NY, PA, RI, VT. †Standard reference population is US standard population during year 2000. ‡Age-specific rates.

### Sex

Death related to blastomycosis was significantly more likely in men than in women (p<0.05). The average age at death was significantly lower for men than for women (p = 0.02) (Table 1). The annual mortality rate over the period obtained from using Poisson regression declined for both men and women (Table 2).

**Table 2:** Age-adjusted time trends in blastomycosis-related mortality rate for sex, race/ethnicity, and geographic region, United States, 1990–2010*

Variable	Annual percent change† in age-adjusted mortality rates (95% CL)	p value
Sex		
F	−2.28 (−3.84, −0.70)	<0.01
M	−2.32 (−3.43, −1.20)	<0.01
Race/ethnicity		
White	−1.57 (−2.62, −0.51)	<0.01
Hispanic	‡	
Black	−5.12 (−7.19, −3.01)	<0.01
Asian	‡	
Native American	‡	
Geographic region		
Northeast	‡	
South	−5.06 (−6.28, −3.28)	<0.01
Midwest	1.70 (0.27, 3.15)	0.02
West	‡	
Total	−2.21 (−3.11, −1.29)	<0.01

*CL, confidence limit.  †Annual percent change based on the Poisson model for death, with year and age group dummy variables as independent variables, and the base population as the offset. No other covariates were adjusted in the model. ‡Few deaths occurred in these categories, and thus, were not included in Poisson regression analyses to avoid inappropriate use of small numbers in Poisson regression modeling.

### Race/Ethnicity

Native Americans and blacks were significantly more likely to die from blastomycosis-related complications than whites; overall, Asians and Hispanics were significantly less likely to die of blastomycosis than other groups (Table 1). The annual mortality rate over the period declined among blacks and whites (Table 2).

### Geographic Region

Most (96.7%) of the blastomycosis-related deaths occurred in the southern and midwestern regions, and a small proportion of deaths occurred in the northeastern and western regions. The midwestern region had the highest mortality rate, followed by the southern, northeastern, and western regions (Table 1). Percentage changes in mortality rates per year over the period, calculated by using Poisson regression, showed an increase in mortality rates in the midwestern region, and a decline in the southern region (Table 2).

Table 3 shows the results of a subanalysis of the demographic characteristics of populations in the southern and midwestern regions. In the southern region, the mean age at death from blastomycosis was significantly lower among Native Americans (p = 0.03), blacks (p<0.01), and Hispanics (p = 0.02) than among whites based on a *t* test for difference in means. In the midwestern region, the mean age at death from blastomycosis was significantly lower among Native Americans (p = 0.02), Asians (p<0.01), blacks (p<0.01), and Hispanics (p<0.01) than among whites. Furthermore, the mean age at death from blastomycosis in the midwestern region was significantly lower among Asians than among Native Americans (p<0.01), blacks (p<0.01), and Hispanics (p = 0.04).

**Table 3:** Demographic distribution and age-adjusted mortality rates for the Midwestern and Southern regions in the United States, 1990–2010

Characteristic	Midwest				South		
	No. (%) deaths	Mean age at death, y	Age-adjusted mortality rate per 1 million person-years (95% CL)		No. (%) deaths	Mean age at death, y	Age-adjusted mortality rate per 1 million person-years (95% CL)
Sex							
F	186 (34.9)	62.7	0.25 (0.21, 0.28)		212 (33.0)	61.7	0.18 (0.16, 0.21)
M	347 (65.1)	60.2	0.57 (0.51, 0.63)		431 (67.0)	59.9	0.46 (0.42, 0.50)
Race/ethnicity							
White	418 (78.4)	64.5	0.34 (0.31, 0.38)		470 (73.1)	64.0	0.29 (0.27, 0.32)
Hispanic	17 (3.2)	47.2	0.45 (0.20, 0.70)		4 (0.6)	50.3	0.02 (0.00, 0.04)
Black	63 (11.8)	50.7	0.55 (0.41, 0.69)		157 (24.4)	50.6	0.46 (0.39, 0.54)
Asian	16 (3.0)	37.3	0.66 (0.29, 1.02)		3 (0.5)	56.7	0.09 (0.00, 0.20)
Native American	19 (3.6)	52.7	0.34 (0.31, 0.38)		9 (1.4)	51.1	0.71 (0.23, 1.20)
Total	533 (100.0)	61.1	0.39 (0.36, 0.42)		643 (100.0)	60.5	0.31 (0.28, 0.33)

## Discussion

Our findings indicate that blastomycosis is a noteworthy cause of preventable death in the United States. These findings confirm the demographic risk factors of blastomycosis indicated in previous case reports and extend these to mortality rates. Blastomycosis death occurred more often among older persons than among younger persons (*24*), and more often among men than women (*2*,*24*). The age association found likely represents waning age-related immune function and higher prevalence of immunocompromising conditions. The observed sex differences in blastomycosis mortality may be attributable to differences in occupational or recreational exposures that increase risk for infection (*14*). For example, those who work outdoors involving construction, excavation, or forestry, or participate in outdoor recreational activities such as hunting (*7**,**11*), may more likely be exposed than those who principally work indoors.

The disproportionate burden of blastomycosis deaths sustained by persons of Native American or black race is also consistent with previous reports (*3*,*24*). Increased exposure and prevalence of infection, reduced access to health care, and genetic differences may play a role in the observed race-specific disparities in blastomycosis mortality rates (*25*). A finding of the current study is that even though persons of Asian descent are at lower risk for dying from blastomycosis than whites, those who died from blastomycosis did so at a much younger age (22.6 years younger). This disparity is even greater in the midwestern region, where Asians died at an age 27.2 years younger than did whites. 

Consistent with the recognized geographic distribution of *B. dermatitidis* (*1**,**2*), we found that death related to blastomycosis occurred more often among persons who resided in the midwestern or southern regions than among those in the western and northeastern regions. During the study period, the southern region showed decreases in mortality rates, and the midwestern region, which had the highest mortality rate, showed an increase in rate.

The use of population-based data and large numbers can provide insight, though some limitations associated with using MCOD data should be considered. First, potential underdiagnosis and underreporting of death related to blastomycosis may lead to underestimates of mortality rates and the true public health burden of blastomycosis in the United States. Low physician awareness of blastomycosis may be a contributor. Second, it was not possible to verify accuracy of recorded data or access supplemental data. For example, there may be reporting errors regarding correct race/ethnicity identification on death certificates and in population census reports. Third, we could not adjust for other possible confounders (i.e., smoking, socioeconomic factors, activity, lifestyle, occupation) because these data are not recorded on death certificates. These limitations must be considered along with our findings. 

This study sheds light on the scope of the incidence of blastomycosis in the United States, though the true incidence may be greater than that reported here. Although *B. dermatitidis* infection may be difficult to prevent because of its widespread distribution in areas where blastomycosis is endemic, deaths resulting from blastomycosis can be prevented with early recognition and treatment of patients with symptomatic infection. The continued incidence of blastomycosis in the United States, as indicated by the observed modest decrease in the mortality rates over the 21-year study period, calls for improvement in provider and community awareness, which may lead to including blastomycosis as a diagnostic consideration in patients with pulmonary disease refractory to treatment. Our findings, recent reports of disproportionately high infection rates among Asians (*26*), and the lack of decline in the mortality rates in the midwestern region support further investigation. We also encourage improvements in blastomycosis surveillance that involve examining trends in incident cases, hospitalization (including length of stay), timely diagnosis, and treatment to further elucidate the burden of blastomycosis in the United States.
